# Unmasking the Failure: Isolation and Cohorting Practice in Low Resource Healthcare Setting.

**DOI:** 10.1017/ash.2025.223

**Published:** 2025-09-24

**Authors:** Fairoze Masuda Akther, Shariful Amin Sumon, Md. Golam Dostogir Harun

**Affiliations:** 1 Infectious Diseases Division, icddr,b, Dhaka, Bangladesh; 2 icddr,b; 3 International Centre for Diarrhoeal Disease Research, Bangladesh (Icddr,b)

## Abstract

**Background:** Isolation and cohorting are essential components of an effective infection prevention and control (IPC) program within healthcare settings to prevent spreading infectious diseases. In Bangladesh, no related study has explored knowledge or practices of isolation and cohorting practice. This study aims to investigate the perception and practice of isolation and cohorting among healthcare workers (HCWs) at tertiary care hospitals in Bangladesh. **Methods:** From September 2020 to January 2021, this hospital-based, multi-center cross-sectional study was conducted in seven tertiary hospitals across Bangladesh. Participants were HCWs (physicians, nurses, cleaning staff) involved in direct patient care and agreed to participate. A pre-tested structured questionnaire was employed through face-to-face interviews for data collection. Descriptive and multivariate analyses were performed using STATA Version 15. Compliance with isolation precautions was categorized into “good” and “poor” groups based on the mean response score. **Results:** A total of 1511 HCWs were interviewed, in the study. Overall, 88.7% of HCWs were familiar with the terms ‘isolation’ and ‘cohorting’. Still, 40.5% of them did not have a thorough understanding of when to implement these strategies. Only 18.0% of the HCWs reported good compliance towards isolation and cohorting practice. Entry-level (under 31 years) HCWs exhibited better compliance to isolation and cohorting compared to mid-level (31 to 40 years) HCWs (59.6% vs 29.4%). The majority of HCWs (93.7%) who participated in infection control training demonstrated a high level of compliance with isolation. In terms of good compliance with isolation precautions, physicians and nurses had higher odds (OR: 11.0) than cleaning staff. Moreover, HCWs with < 5 years of experience were more likely to comply with good adherence (OR: 1.56, 95%CI: 1.09-2.21) than those with over 10 years of experience. **Conclusions:** The study revealed that HCWs have a limited understanding and implementation of isolation and cohorting practices. To address this issue, policymakers and hospital leadership should adopt strategies that promote these practices by providing regular training and awareness programs for healthcare workers. Such initiatives are essential to help limit the spread of infections in healthcare settings.

